# Disentangling complex genomic signals to understand population structure of an exploited, estuarine‐dependent flatfish

**DOI:** 10.1002/ece3.8064

**Published:** 2021-08-30

**Authors:** Shannon J. O'Leary, Christopher M. Hollenbeck, Robert R. Vega, David S. Portnoy

**Affiliations:** ^1^ Department of Biology Saint Anselm College Manchester New Hampshire USA; ^2^ Marine Genomics Laboratory Department of Life Sciences Texas A&M University Corpus Christi Corpus Christi Texas USA; ^3^ CCA Marine Development Center Texas Parks and Wildlife Department Corpus Christi Texas USA

**Keywords:** genetic‐environmental association, local adaptation, population genomics

## Abstract

Interpreting contemporary patterns of population structure requires an understanding of the interactions among microevolutionary forces and past demographic events. Here, 4,122 SNP‐containing loci were used to assess structure in southern flounder (*Paralichthys lethostigma*) sampled across its range in the US Atlantic Ocean (Atlantic) and Gulf of Mexico (Gulf) and relationships among components of genomic variation and spatial and environmental variables were assessed across estuarine population samples in the Gulf. While hierarchical amova revealed significant heterogeneity within and between the Atlantic and Gulf, pairwise comparisons between samples within ocean basins demonstrated that all significant heterogeneity occurred within the Gulf. The distribution of Tajima's *D* estimated at a genome‐wide scale differed significantly from equilibrium in all estuaries, with more negative values occurring in the Gulf. Components of genomic variation were significantly associated with environmental variables describing individual estuaries, and environment explained a larger component of variation than spatial proximity. Overall, results suggest that there is genetic spatial autocorrelation caused by shared larval sources for proximal nurseries (migration/drift), but that it is modified by environmentally driven differentiation (selection). This leads to conflicting signals in different parts of the genome and creates patterns of divergence that do not correspond to paradigms of strong local directional selection.

## Graphical abstract

4,122 SNP‐containing loci were used to assess structure in southern flounder (*Paralichthys lethostigma*) sampled across its range in the U.S. Atlantic Ocean (Atlantic) and Gulf of Mexico (Gulf). Components of genomic variation were significantly associated with environmental variables describing individual estuaries, and environment explained a larger component of variation than spatial proximity. In sum, results suggest that there is genetic spatial autocorrelation caused by shared larval sources for proximal nurseries (migration/drift), but that it is modified by environmentally driven differentiation (selection).

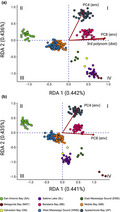

## 1 INTRODUCTION

Patterns of contemporary genetic variation within and among populations result from interactions among microevolutionary forces (i.e., genetic drift, migration, selection, and mutation) against the backdrop of historical demographic changes, including population expansion and bottlenecks (Luikart et al., 2003). Because these forces result in allele frequency changes at specific loci (e.g., selection) and across loci in the genome as a whole (e.g., drift), studies employing dense genome‐wide sampling of genetic markers have become the gold standard for understanding and interpreting current patterns of population structure in exploited marine species (Bernatchez et al., 2017). Central to this endeavor is a model, supported by both population genetic theory and empirical studies, that posits strong geographically localized directional selection which will create elevated divergence with respect to the rest of the genome and that these areas of increased divergence (outlier loci) should be confined to a small proportion of the genome (Lewontin & Krakauer, 1973; Nielsen, 2001).

While this paradigm is important, other factors may confound interpretation, including selection pressures changing with ontogeny, polygenic traits, violations of equilibrium assumptions, and complex interactions of other microevolutionary forces (Forester et al., 2018; Hoban et al., 2016; Lotterhos & Whitlock, 2014). Consideration of such factors may be particularly important for marine bony fishes, which frequently feature complex life histories in which the larval phase differs greatly in size, behavior, and other aspects of basic biology from the juvenile and adult phases. While juveniles are more similar to adults than larvae, they usually occupy different trophic levels and have different habitat requirements than adults. Habitat changes may occur several times during development, suggesting ontogenetic shifts in selective pressures are likely (Dahlgren & Eggleston, 2000; Yang et al., 2018). Additionally, many marine species have large population sizes and high fecundity (Hedgecock & Pudovkin, 2011). This results in a decrease of the relative strength of genetic drift while increasing the opportunity for adaptive variants to arise via mutation (Cormack et al., 1990). Further, marine species are frequently geographically widespread across heterogeneous environments, leading to opportunities for localized selection to create adaptive genetic variation underlying phenotypic traits including life history, morphology, behavior, and physiology (Bernatchez, 2016). Frequently, these are polygenic traits, though some complex traits may also be controlled by a single locus or chromosomal region (Prince et al., 2017). High degrees of connectivity due to larval dispersal and adult migration are typical of marine species and while theoretically this could weaken the effects of selection (Felsenstein, 1976), studies have shown that marine species often display local adaptation despite high gene flow (Clarke et al., 2010; Hoey & Pinsky, 2018). Taken together, the combination of these factors could lead to a scenario where selection acts upon different genes and regions of the genome with a wide range of effect sizes (Gagnaire & Gaggiotti, 2016) that are not always detectable using outlier detection (genome scan) methods (Bernatchez, 2016; Gagnaire & Gaggiotti, 2016). Finally, for many marine species, historical changes in climate and sea level have led to fluctuations in population size and connectivity (Marko & Hart, 2012; Portnoy et al., 2014), which may lead to remnant historical demographic signals still present in the genome.

Southern flounder, *Paralichthys lethostigma*, inhabit estuarine and nearshore environments along the U.S. Atlantic coast (Atlantic) from the Carolinas to Florida and across the northern Gulf of Mexico (Gulf) to near Veracruz in Mexican waters, with a break in distribution along the southern Florida peninsula. They support substantial commercial and recreational fisheries, accounting for a multi‐million‐dollar fishery in U.S. waters of the Atlantic and northern Gulf (Flounder Technical Task Force, 2015). Long‐term declines in abundance of young‐of‐the‐year (YOY), juvenile, and adult southern flounder are well documented in the Gulf and have been attributed to fishing mortality (Flounder Technical Task Force, 2015; Froeschke et al., 2011). Recently, steep declines in the number of YOY in the western Gulf have prompted an interest in a stock‐enhancement program where hatchery‐reared YOY, spawned from captive wild‐caught adults, are used to augment recruitment (Kaiser et al., 2012; Miller et al., 2010). Both the high market value of southern flounder and its suitability as a target for commercial and stock augmentation (Daniels et al., 1996; Watanabe et al., 2006) warrant a robust assessment of population structure.

Southern flounder have a high dispersal potential resulting from larval dispersal, ontogenetic shifts in habitat use, and adult movement. Adults reside in bays and estuaries for much of the year but migrate to spawning grounds located in multiple offshore locations in the fall. This provides opportunity for gene flow as spawning grounds do not necessarily correspond to individual estuaries of origin for adults (Craig et al., 2015). In addition, individual estuaries may receive larval subsidies from more than one spawning ground as buoyant eggs are transported to nursery habitats where larvae settle in shallow estuarine and freshwater environments in late winter and early spring (Nims & Walther, 2014). Larval supply to specific estuaries is affected by multiple processes, including currents, tides, and hydrography of individual estuaries. In addition, long larval duration (30–60 days) results in the potential for long‐range dispersal (Bailey et al., 2005). Southern flounder YOY exhibit shifts in habitat preference as they grow, and different size/age classes are found in different microhabitats within bays and estuaries, characterized by different levels of salinity and differences in substrate and vegetation (Furey & Rooker, 2013; Glass et al., 2008; Nañez‐James et al., 2009). Juveniles remain in the same estuary until maturity at approximately two years of age, and tagging studies indicate limited adult movement during seasonal estuarine residence (Craig et al., 2015; Monaghan, 1992). Analyses of otolith microchemistry suggest a lack of nursery‐site fidelity among adults (Wang et al., 2018), and little is known about habitat use during and after offshore spawning. Additionally, there is evidence that some adults may remain permanently offshore (Watterson & Alexander, 2004).

Previous studies of stock structure based on allozymes (Blandon et al., 2001), mitochondrial DNA (Anderson et al., 2012), microsatellites (Wang et al., 2015), and otolith morphometrics (Midway et al., 2014) have identified differences between southern flounder in the Atlantic and Gulf but failed to demonstrate differences within each region. By contrast, regional and estuarine‐specific differences in life‐history traits such as growth rates and age/size‐at‐maturity have been documented, suggesting the potential for genetic differences at sufficiently small spatial‐scales that microsatellites and mtDNA cannot resolve (Corey et al., 2017; Fischer & Thompson, 2004). Additionally, heterogeneity in habitat within and among estuaries inhabited by southern flounder may impact growth and survival at multiple life stages (Corey et al., 2017; Midway et al., 2015). This heterogeneity includes differences in habitat availability, levels of freshwater input, substrate types, hydrography, temperature, salinity, nutrient loading, and suspended sediment (NEAA, 2018). Variance in environmental conditions across estuaries could create differences in local selection pressures thus leading to changes in components of genetic variation that could persist despite gene flow or even contribute to the disruption of gene flow (Tigano & Friesen, 2016). Finally, historic events are important to shaping contemporary genomic variation. For example, population expansion after glacial periods have been demonstrated for multiple marine taxa in the Atlantic and Gulf (Marko & Hart, 2012; Portnoy et al., 2014) and are likely important for southern flounder as well.

To understand the relative roles that environment, geography, and demographic history have played in shaping contemporary population structure of southern flounder, reduced representation DNA sequencing was used to examine genomic variation of the southern flounder genome from individuals sampled throughout their range. By using several thousand loci randomly distributed throughout the genome, finer‐scale patterns of population structure can be resolved as compared with previous data sets consisting of tens of loci (mtDNA/microsatellite studies). Additionally, these data sets consist of loci in the coding and noncoding parts of the genome, enabling an assessment of population structure using both neutral and presumably adaptive genetic variation and screening for associations among components of genomic variation and environmental variables. Typically, multiple single‐nucleotide polymorphisms (SNPs) are detected on single DNA fragments generated using next‐generation sequencing. To reduce effects of linkage, these SNPs need to be thinned. Instead, a haplotype‐based approach was implemented, generating a data set consisting of multiallelic loci, and thus combining both the power of a larger number of loci and multiple alleles per locus (Baetscher et al., 2018; Willis et al., 2017). Additionally, this approach allowed for the implementation of DNA sequence‐based analyses to test for conformance to equilibrium assumptions at individual locus and genome‐wide scales indicative of selection/local adaptation versus population expansion, respectively.

## 2 METHODS

### 2.1 Sampling design

**FIGURE 1:**
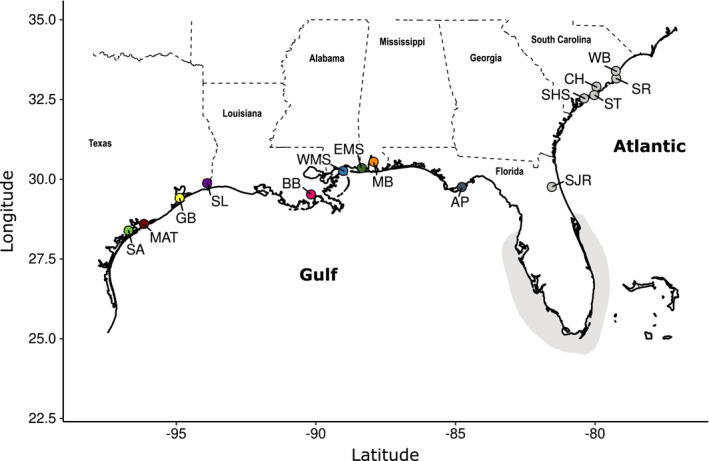
Sample distribution of southern flounder young‐of‐the‐year, juveniles, and adults sampled in estuaries throughout the Gulf of Mexico and Western Atlantic. Sample locations from West to East and South to North are San Antonio Bay (SA, *N* = 23), Matagorda Bay (MAT, *N* = 3), Galveston Bay (GB, *N* = 2), Sabine Lake (SL, *N* = 24), Barataria Bay (BB, *N* = 39), West Mississippi Sound (WMS, *N* = 34), East Mississippi Sound (EMS, *N* = 5), Mobile Bay (MB, *N* = 62), Apalachicola Bay (AP, *N* = 44), St. John's River (SJR, *N* = 20), St. Helena Sound (SHS, *N* = 4), Stono‐North Rivers (*N* = 4), Charleston Harbor (CH, *N* = 18), Santee Rivers (SR, *N* = 4), and Winyah Bay (WB, *N* = 18). Sample locations in the Gulf are colored to match Figures 4 and 5. The approximate extent of the break in distribution off the coast of Florida is indicated in grey

Tissues (fin clips) were taken from YOY, juveniles, and adults sampled from six estuaries in the Atlantic and nine estuaries in the Gulf (Figure 1, Table 1) during regularly occurring surveys by the Florida Fish and Wildlife Conservation Commission, Texas Parks and Wildlife Department, and South Carolina Department of Natural Resources from 2013 to 2016. Because individuals remain in their natal estuaries for approximately two years, attempts were made to obtain exclusively YOY (defined as individuals caught at <25 cm) from each year to determine estuary of origin. Drastic declines in the number of YOY caught during regular surveys did not allow for sufficient sample sizes. Instead, sample sizes were supplemented with juveniles (25–40 cm) and adults (>40 cm) caught in or near estuaries. Small sample sizes for each cohort did not allow for a formal test of cohort effects and assignment of adults to estuaries, though exploratory analysis restricting the data set to only YOY caught in the same year yielded the same overall patterns as analysis with mixed cohorts, indicating that cohort effects are negligible. In addition, mixed age classes should help to minimize temporal effects caused by recruitment variation. Therefore, samples were pooled across life stages during formal data analysis.

**TABLE 1:** Sample size per estuary and age class. Young of the year (YOY) defined as fish caught at 25 cm or less, juveniles as 25–40 cm and adults as >40 cm

Ocean basin	Estuary	Adults	Juveniles	YOY
Gulf	Apalachicola Bay	1	3	40
	Barataria Bay	7	32	n/a
	Charleston Harbor	7	9	2
	East Mississippi Sound	3	2	n/a
	Galveston Bay	n/a	2	n/a
	Matagorda Bay	n/a	n/a	3
	Mobile Bay	48	14	n/a
	Sabine Lake	2	19	3
	San Antonio Bay	3	10	10
Atlantic	Santee Rivers	2	2	n/a
	St Helena Sound	2	2	n/a
	St John's River	n/a	n/a	20
	Stono‐North Edisto Rivers	1	n/a	3
	West Mississippi Sound	9	23	2
	Winyah Bay	7	8	3

### 2.2 Genotyping

DNA was extracted using Mag‐Bind Blood and Tissue DNA kits (Omega Bio‐Tek). Double digest restriction site‐associated DNA (ddRAD) libraries were constructed using a modified protocol (Portnoy et al., 2015) and sequenced on four separate lanes of an Illumina HiSeq 2500. Raw sequences were demultiplexed using *process_radtags* (Catchen et al., 2011). Quality trimming, read mapping, and SNP calling were performed using the *dDocent* pipeline (Puritz et al., 2014) and a reduced‐representation reference genome (approximately 2%–5% of the genome) previously produced for southern flounder (O'Leary et al., 2018). Raw SNPs were rigorously filtered for quality following recommended practices (O'Leary et al., 2018). Quite commonly, more than one SNP was identified on a single DNA fragment, and rather than thin SNPs to retain only one biallelic SNP per contig, *rad_haplotyper* (Willis et al., 2017) was used to collapse SNPs on the same contig into haplotypes, producing a data set consisting of 4,122 multiallelic loci (2–80 alleles per locus). Data analysis was primarily allele frequency‐based, apart from the test of neutrality using Tajima's *D*, which requires DNA sequences to test for mutation‐drift equilibrium. Detailed processing steps, reproducible code, and threshold values used are available from https://github.com/sjoleary/SFL_PopGen.

### 2.3 *F*_ST_‐outlier analysis

Presence of *F*_ST_‐outlier loci was assessed using two methods: the fdist‐method implemented in arlequin (Excoffier & Lischer, 2010), and the Bayesian approach used in bayescan (Foll & Gaggiotti, 2008). For both methods, outlier loci with significantly higher *F*_ST_ values than expected under a neutral model are flagged as loci putatively under directional selection. Given low background *F*_ST_ values typically found in marine fishes (Waples, 1998), an assessment for loci putatively under balancing selection (*F*_ST_ significantly lower than expected) was not conducted. Analysis in arlequin was based on 20,000 coalescent simulations, using a strict island model. To account for multiple testing, *p*‐values were corrected according to Benjamini and Hochberg (1995). bayescan runs consisted of 25 pilot runs of 5,000 iterations, followed by a total of 550,000 iterations (burn‐in of 50,000 iterations, 10,000 samples with a thinning interval of 50) a *q*‐value of .05 was used as the threshold for outlier detection. For both methods, *F*_ST_‐outlier analysis was run using all individuals grouped by estuary and then using individuals grouped by estuary separately for each ocean basin. The distribution of loci flagged during *F*_ST_‐outlier analysis across linkage groups (chromosomes) was assessed using a previously established linkage map (O'Leary et al., 2018).

### 2.4 Assessment of population structure and genetic diversity

Loci were subdivided into two datasets: outlier (consensus loci identified by both outlier detection methods) and neutral loci (all remaining loci). Hierarchical, locus‐by‐locus analysis of molecular variance (amova), as implemented in arlequin, was used to test for homogeneity of genetic variation both between basins and among estuaries within basins. Homogeneity within each basin was explored for neutral loci further using a single‐level amova. For amova, the significance of each component of variation was assessed by permuting individuals between groups 10,000 times. Pairwise estimates of *F*_ST_ were also generated in arlequin, as a post hoc test for homogeneity between estuaries. Significance was determined as above and correction for multiple comparisons applied following Benjamini and Hochberg. Only samples from estuaries with ≥18 individuals were used in the analysis. Although 20–30 individuals has been suggested as the minimum sample size for reliable allele frequency estimates, choosing 18 samples allowed for balance of retaining as many groups as possible while providing sufficient power (Luikart & Cornuet, 1998; Morin et al., 2009).

Genomic diversity of each estuary was determined as Nei's gene diversity (Nei, 1973), rarefied allele counts, and evenness. The last is a measure of the distribution of allele frequencies and was estimated as the ratio of the Stoddart & Taylor index (diversity weighted for more abundant alleles) and the Shannon‐Wiener index (diversity weighted for rarer alleles), as implemented in *poppr* (Kamvar et al., 2014). For each measure of diversity, a Friedman's rank sum test was used to test homogeneity among estuaries. A Wilcoxon signed‐rank test was used post hoc to test for pairwise differences between estuaries; *p‐values* were corrected for multiple comparisons according to Benjamini and Hochberg. The number of fixed loci was documented for each ocean basin and estuary and the global rarefied allele counts for loci fixed in a given group of individuals compared.

### 2.5 Tests of neutrality at the genome and locus level

The observed genome‐wide distribution of Tajima's *D* was calculated for each estuary and compared with a null‐distribution of an equivalent set of loci in drift‐mutation equilibrium. While significant deviations from equilibrium at individual loci are indicative of selection, a genome‐wide deviation indicates that demographic events (population expansion/bottlenecks) have occurred. This analysis made use of the fact that DNA sequence information can be retrieved from microhaplotypes (SNP‐containing loci). The population‐scaled mutation rate Θ was estimated for each locus as the average number of pairwise differences per site between all pairs of microhaplotypes (Θ_T_, nucleotide diversity; Nei, 1987), and as the number of segregating sites across all microhaplotypes (Watterson's estimator Θ_W_, Watterson, 1975). In an equilibrium population of stable size and without selection, Θ_T_ will approximate Θ_W_, but when a population has undergone recent expansion or there is strong direction selection, Θ_W_ will exceed Θ_T_ because the latter is sensitive to allele frequencies and the former is not. Tajima (1989) formalized this observation in a test statistic, Tajima's *D*, that can be used to test for conformance to drift‐mutation equilibrium assumptions.

Tajima's *D* was calculated for each locus with individuals grouped by estuary, creating a set of estuary‐specific observed distributions. Then, 1,000 genome‐wide, null distributions of Tajima's *D* were simulated for each estuary, using a coalescent model, executed in MS (Hudson, 2002). To do this, a set of neutral loci consisting of the same number of loci with the same distribution of segregating sites as in the observed data (grouped by estuary) was generated. For example, if 100 loci in the empirical data set have four segregating sites, the simulated data set also contains 100 loci with four segregating sites. The difference between observed and simulated distributions was then assessed for each estuary by comparing mean and median values of the empirical distribution with those of the simulated distributions. Significance was assessed by determining the proportion of times that the observed value was smaller or larger than simulated values.

Further, locus‐specific effects were assessed by testing for significant deviation from neutrality as implemented in *pegas* (Paradis, 2010); *p*‐values were corrected by estuary for each locus according to Benjamini and Hochberg (1995) to account for multiple comparisons. The number of significant (*p* < .05) loci, positive and negative, was then summarized by estuary. The distribution of loci with significant Tajima's *D* values across linkage groups was assessed using only those loci that were previously incorporated into a linkage map (O'Leary et al., 2018).

Finally, to better understand what was driving patterns in neutrality tests, Θ was calculated based on the number of segregating sites as Θ_W_ (Watterson, 1975) and based on pairwise differences among haplotypes as Θ_T_ (nucleotide diversity; Nei, 1987), as implemented in *pegas*, and the mean and standard deviation compared across estuaries. A Mann–Whitney test was used to test for a significant difference in mean Θ‐values across estuaries and between ocean basins.

### 2.6 Landscape genetics

Redundancy analysis (RDA), as implemented in *vegan* (Oksanen et al., 2013), was used to disentangle the influence of geographic distance and environmental variables and assess their interaction on observed patterns of genomic variation among samples from the Gulf. RDA was not carried out among samples from the Atlantic because of the limited number of geographic samples with enough individuals (3) and their uneven, limited geographic spread (see Figure 1). RDA is a constrained ordination method that extracts and summarizes components of variation in a multidimensional data set explained by a set of explanatory variables. It is a useful approach when using genomic data, that does not rely on equilibrium assumptions present in *F*_ST_‐based analyses (Forester et al., 2018). The *R* ^2^ value can be understood as the proportion of genomic variation explained by constraining variables, allowing for a comparison of the relative importance of these variables and their interaction. Here, the RDA was used to parse and visualize components of genomic variation (response variables) that are explained by geography and/or environment (constraining variables) and to identify alleles/loci driving any observed environmental pattern. To achieve this, two constraining matrices were generated, one describing spatial patterns and one describing environmental differences among estuaries. For each, forward selection was used to identify the best model using *R* ^2^ as the stopping criterion (Oksanen et al., 2013). Geographic distance was measured as the approximate coastline distance between mouths of estuaries; distances were jittered for individual fish to account for individual variability in sample location. Data for 39 environmental variables for each of the included sample locations in the Gulf were obtained from the National Estuary Eutrophication Assessment database (NEAA, 2018). This data set was chosen as it includes all estuaries being evaluated and contains a broad range of variables describing the long‐term hydrology; abiotic variables such as nutrients, temperature, salinity, pH, or suspended particles; and climate data, for example, humidity, precipitation, or wind speed. Because the goal was to enumerate environmental differences among estuaries, not necessarily to identify individual environmental pressures (for the latter environmental data collected concurrent to YOY residence in bays/estuaries would be more appropriate), variables were PCA‐transformed, resulting in new synthetic variables summarizing environmental differences among estuaries. After identifying the best models describing components of genetic variation explained by spatial and environmental models alone, variance partitioning was used to compare the contribution of geographic distance and environmental differences in structuring observed genomic variation and disentangle whether geography or the environment plays a larger role in shaping genetic diversity. A full model, using geographic and PCA‐transformed environmental variables, a partial model using geographic data conditioned on environmental variables, and a partial model using environmental variables conditioned on geographic data, were considered to partition the explainable variance into individual (geography or environment) and shared components (geography plus environment), using *vegan*. Significance of each component was tested using 1,000 permutations. The environmental model was then used to identify loci most strongly associated with environmental differences among estuaries. Alleles with a Mahalanobis distance >25 on the first two RDA‐axes were flagged as most strongly associated with PCA‐transformed environmental variables. The distribution of alleles (loci) having a Mahalanobis distance >25 across linkage groups was assessed using those loci that previously were incorporated into a linkage map (O'Leary et al., 2018).

All figures were generated using *ggplot2* (Wickham, 2009) and *UpsetR* (Conway et al., 2017). An Rmarkdown and corresponding rendered html‐document containing reproducible code for the complete analysis and functions as a standalone extended presentation of methods and results can be accessed at https://github.com/sjoleary/SFL_PopGen.

## 3 RESULTS

### 3.1 *F*_ST_‐outlier analysis

The final filtered data included 316 individuals from six estuaries in the Atlantic and nine estuaries in the Gulf (Table 1, Figure 1). No *F*_ST_‐outlier loci were detected using either approach when Gulf or Atlantic individuals were assessed separately.

### 3.2 Assessment of population structure and genetic diversity

Hierarchical amova implemented to test for heterogeneity between and within ocean basins revealed significant divergence (*p* < .0001) between the Atlantic and Gulf in both neutral and outlier loci (Table 2). The magnitude of *F*_CT_ differed between the two data sets: 0.0414 (neutral loci) and 0.3275 (outlier loci). Further, divergence in neutral loci among estuaries within ocean basins was significant (*F*_SC_ = 0.0016, *p* = .0093), whereas divergence in outlier loci was not (*F*_SC_ = 0.0020, *p* = .6020). Divergence among estuaries using neutral loci only was significant in the Gulf (*F*_ST_ = 0.0014, *p* = .027,) but not in the Atlantic (*F*_ST_ = 0.0026, *p* = .098, Table 2). By contrast, all pairwise comparisons between estuaries in different ocean basins were significant (*p* < .001) for both neutral and outlier loci (Table 3). For neutral loci, comparison of San Antonio Bay (SA) and Sabine Lake (SL) was significant before (but not after) correction for multiple tests, while San Antonio Bay (SA) and Apalachicola Bay (AP) remained significantly different even after correction for multiple tests. All remaining comparisons between estuaries within ocean basins were nonsignificant for both neutral and outlier loci (Table 3).

**TABLE 2:** Locus‐by‐locus AMOVA using only (A) neutral and (B) outlier loci indicating variance partitioning, *F*‐statistic, and significance of each component

Source of variation	Percentage variation	Average *F*‐statistic over all loci	*p*‐Value
(A)			
Among oceans	4.1348	0.0414	<.0001*
Among estuaries within oceans	0.1551	0.0016	.0093*
Among individuals within estuaries	95.7101	0.0429	<.0001*
(B)			
Among oceans	32.7517	0.3275	<.0001*
Among estuaries within oceans	0.1332	0.0020	.6020
Among individuals within estuaries	67.1150	0.3289	<.0001*

Note

Significant values are in bold and marked with *.

**TABLE 3:** Comparison of pairwise *F*_ST_ (above the diagonal) and level of significance (below the diagonal) between all pairs of estuaries in the Gulf and Atlantic using (A) neutral and (B) outlier loci only

	SA	SL	BB	WMS	MB	AP	SJR	CH	WB
(A)									
SA	–	0.0020	0.0010	0.0014	0.0011	0.0016*	0.0466*	0.0423*	0.0443*
SL	0.0329	–	0.0010	0.0010	0.0004	0.0010	0.0452*	0.0411*	0.0427*
BB	0.2101	0.3244	–	0.0006	0.0007	0.0007	0.0462*	0.0421*	0.0439*
WMS	0.0644	0.3757	0.3804	–	0.0004	0.0005	0.0462*	0.0420*	0.0441*
MB	0.1253	0.9022	0.0876	0.6603	–	0.0006	0.0450*	0.0405*	0.0428*
AP	0.0040	0.2847	0.1480	0.5679	0.2009	–	0.0457*	0.0414*	0.0432*
SJR	0.0000	0.0000	0.0000	0.0000	0.0000	0.0000	–	0.0010	0.0011
CH	0.0000	0.0000	0.0000	0.0000	0.0000	0.0000	0.4436	–	0.0011
WB	0.0000	0.0000	0.0000	0.0000	0.0000	0.0000	0.6043	0.1469	–
(B)									
SA	–	−0.0011	0.0019	0.0046	−0.0002	0.0021	0.3399*	0.3419*	0.3055*
SL	0.8747	–	0.0006	0.0050	0.0017	0.0014	0.3419*	0.3445*	0.3063*
BB	0.4627	0.6881	–	0.0008	0.0017	0.0026	0.3480*	0.3436*	0.3101*
WMS	0.1503	0.1274	0.5995	–	0.0019	0.0035	0.3265*	0.3244*	0.2906*
MB	0.7789	0.3626	0.2657	0.2619	–	0.0015	0.3400*	0.3347*	0.3032*
AP	0.3948	0.5244	0.1920	0.1163	0.2855	–	0.3481*	0.3439*	0.3122*
SJR	0.0000	0.0000	0.0000	0.0000	0.0000	0.0000	–	−0.0077	0.0008
CH	0.0000	0.0000	0.0000	0.0000	0.0000	0.0000	0.9928	–	−0.0003
WB	0.0000	0.0000	0.0000	0.0000	0.0000	0.0000	0.6027	0.6214	–

Note

Significant values are in bold and marked with *.

**FIGURE 2:**
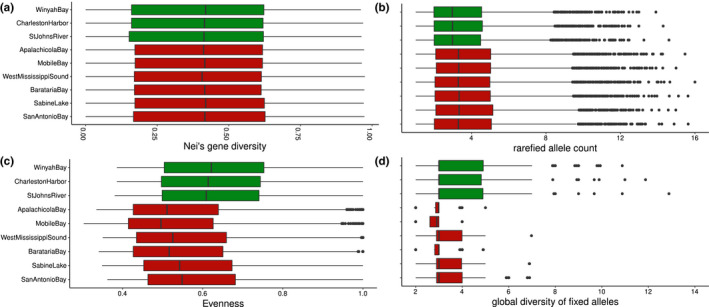
Distribution of (a) Nei's gene diversity, (b) rarefied allele count, (c) evenness, and (d) global diversity of fixed alleles. Estuaries in the Gulf and Atlantic are represented in red and green, respectively

**FIGURE 3:**
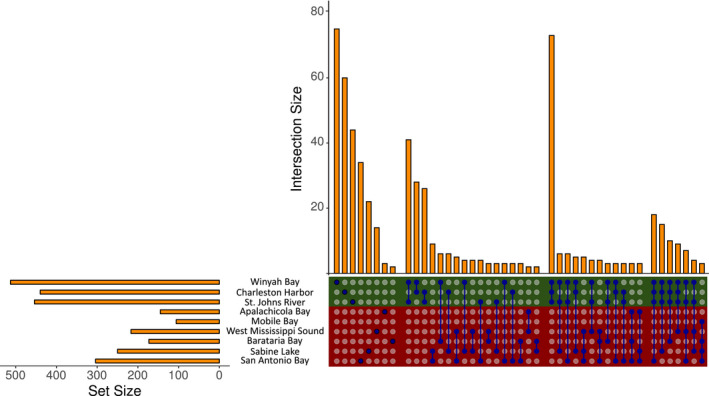
Comparison of fixed loci across sampled estuaries in the Gulf (red box) and Atlantic (green box). The set size (horizontal orange bars) indicates the total number of loci fixed in a given location, the intersection size (vertical orange bars) indicates the number of loci fixed only in a single location (single blue dot) or in two, three, or four locations (indicated by blue dots connected by line)

The comparison of genomic diversity among estuaries revealed significant heterogeneity among estuaries for all three metrics of within‐group genetic diversity: Nei's gene diversity (*Q*_[8]_ = 158.21, *p* < .0001), rarefied allele counts (*Q*_[8]_ = 1,536.5, *p* < .0001), and evenness (*Q*_[8]_ = 3,842.3, *p* < .0001, Figure 2). Nei's gene diversity and rarefied allele counts were significantly higher in estuaries in the Gulf for all 18 pairwise comparisons with estuaries in the Atlantic (Figure 2a,b). All comparisons of gene diversity between estuaries within basins were nonsignificant except for comparisons with Sabine Lake, which had significantly higher gene diversity than all other estuaries in the Gulf (Figure 2b). Similarly, all comparisons of rarefied allele counts between estuaries within basins were nonsignificant except for comparisons with Sabine Lake, which had significantly higher allele counts than all other estuaries in the Gulf, and Mobile Bay which had significantly higher allele counts than West Mississippi Sound (Figure 2b). Evenness was significantly lower in estuaries in the Gulf for all pairwise comparisons with estuaries in the Atlantic (Figure 2c) and no significant differences were found within basins. A comprehensive table with all pairwise tests for all three metrics is found in Table S1. Finally, there was a higher frequency of fixed alleles in estuaries in the Atlantic as compared with the Gulf (Figure 3). A comparison of the intersection of sets of fixed alleles indicated that the largest intersects were one‐set intersects, that is, loci fixed in a single Atlantic estuary (44–75), a three‐set intersect of loci fixed in all three Atlantic estuaries (73) and two‐set intersects consisting of combinations of two Atlantic estuaries (Figure 3). In general, loci fixed in Gulf estuaries had low global diversity, while loci fixed in the Atlantic were a mixture of loci with low and high global diversity (Figure 2d).

### 3.3 Tests of genome and locus neutrality

The genome‐wide distribution of Tajima's *D* was determined for each estuary and compared with an equivalent simulated data set consisting of loci in neutral, drift‐mutation equilibrium to test for patterns consistent with past demographic events. For each estuary, the observed genome‐wide distributions of Tajima's *D* values were left‐shifted relative to simulated equilibrium distributions and observed mean and median values of Tajima's *D* were significantly more negative than the simulated values for each estuary (*p* < .001; Table S2). This pattern is consistent with population expansion.

Locus‐specific effects were assessed by testing for the significant deviation from mutation‐drift equilibrium for each locus for individuals grouped by estuary. A total of 422 loci (10.2% of all loci examined) were significantly different from the expectation of equilibrium (*p* < .05) in at least one estuary; after accounting for multiple comparisons 12 remained significant: 63 (5) were positive, 361 (9) were negative, and 167 (2) were positive in at least one estuary and negative in at least one other estuary. The number of significant positive loci ranged from 15 to 21 across estuaries in the Atlantic; after adjusting for multiple comparisons Winyah Bay and Charleston Harbor had one negative outlier each, while none were found in St John's River individuals, compared from 11 to 19 across estuaries in the Gulf (2–4 after adjusting *p‐values*). The number of significant negative loci across estuaries in the two ocean basins differed markedly; estuaries in the Gulf averaged 91.7 significant negative loci (range = 80–108; 1–4 after adjustment), whereas estuaries in the Atlantic averaged 25.3 (range = 20–31); after adjusting for multiple comparisons, Charleston Harbor and St John's River each had one significant outlier while none were observed in Winyah Bay. A total of 155 loci with significant positive (52), or negative (101), or both (2) Tajima's *D* values in at least one estuary had been mapped previously and appeared to map randomly across all 24 linkage groups (Figure S1), suggesting no specific regions of the genome were in disequilibrium.

Tajima's *D* is derived from a comparison of the number of segregating sites and the average pairwise difference among all sequences, and to understand the mechanisms contributing to departure from mutation drift‐equilibrium these two metrics were compared. Mean values of Watterson's estimator Θ_W_ (based on the number of segregating sites) were significantly higher and more variable in Gulf estuaries (Θ_W_ = 0.454–0.602) as compared with Atlantic estuaries (Θ_W_ = 0.353–0.370, Table S3). By contrast, mean values for Θ_T_ (based on pairwise differences among haplotypes) had a much smaller range (Θ_T_ = 0.0020–0.0021, Table S4) across all estuaries. Sabine Lake had significantly higher Θ_T_ than all other bays, while Winyah Bay displayed the opposite pattern (Table S4). Mann–Whitney tests indicated that both Θ estimates were significantly higher in the Gulf compared with the Atlantic (*P*(Θ_W_) = 0.0282, *P*(Θ_T_) = 0.0282).

### 3.4 Landscape genomics

**FIGURE 4:**
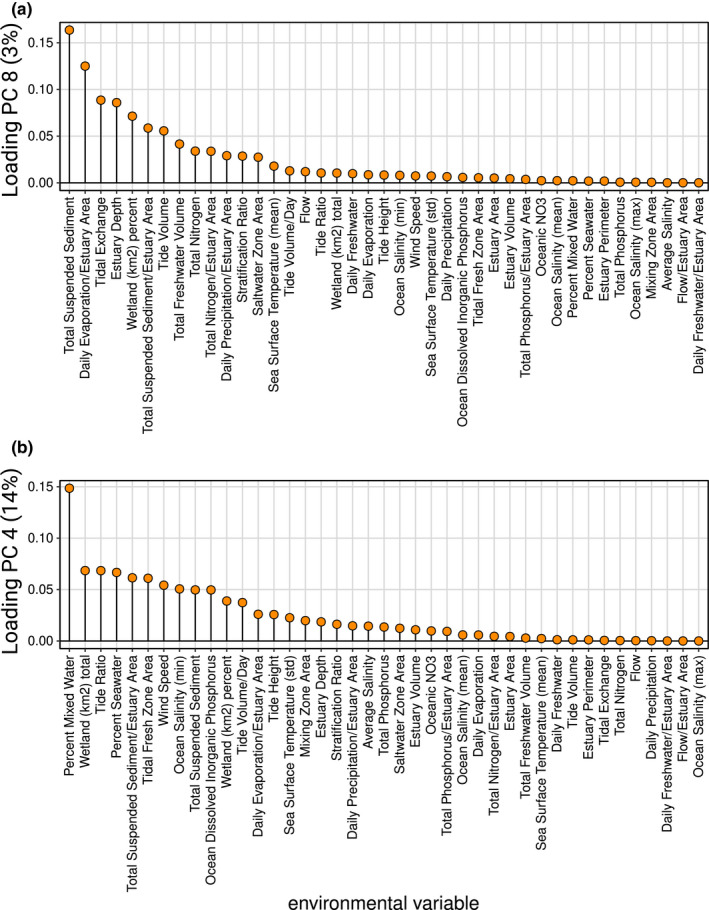
Loading plots for principal component analysis of environmental variables describing sampled estuaries for (a) principal component 8 and (b) principal component 4 which explain 3% and 14% of environmental differences among estuaries, respectively

The RDA framework was used to test for the effects of geographic distance and environmental differences among estuaries in the Gulf on genomic diversity, to identify loci strongly associated with environmental differences, and to partition the components of variance associated with distance, environment, and their interactions. The selected model for geographic distance included the third polynomial of coastal distance, indicative of a pattern of nonlinear isolation by distance. The selected model for environmental differences among estuaries included principal components eight and four. Loading plots for both principal components (Figure 4) indicated that parameters with largest impact include variables related to freshwater inflow (e.g., total suspended sediment, total freshwater volume), tidal influence (e.g., tidal exchange, estuary depth, tide volume, tide ratio), factors determining differences in salinity within and among estuaries (daily evaporation, freshwater volume, percent mixed water, percent seawater), and amount of available wetland habitat.

After determining the best models describing spatial and environmental patterns across estuaries, variance partitioning was performed to evaluate relative impacts of spatial patterns compared with environmental differences. The full model that included both the selected spatial and environmental model was significant (*p* = .023), though it explained only a small proportion of variance in the total genomic data set (1.3%, adjusted *R* ^2^ = .00023). The largest component of variance was explained by environmental variables and shared effects (adjusted *R* ^2^ = .00026, *p* = .005), while the component of spatial, environmental, and shared effects was marginally lower (adjusted *R* ^2^ = .00024, *p* = .029). The smallest significant component of the full model was spatial and shared effects (adjusted *R* ^2^ = .00014, *p* = .020, Table 4).

**TABLE 4:** Partitioning of variance explained by costal distance (xy), environmental variables (env) and shared effects (shared) due to interaction of distance and environment

Partition	Variance (adjusted *R* ^2^)	*p*‐Value
residuals	0.99976	n/a
env + shared	0.00026	.005*
env + xy + shared	0.00024	.029*
shared	0.00016	n/a
xy + shared	0.00014	.020*
env	0.00010	.200
xy	−0.00002	.560

Note

Significant values in bold and marked with *.

**FIGURE 5:**
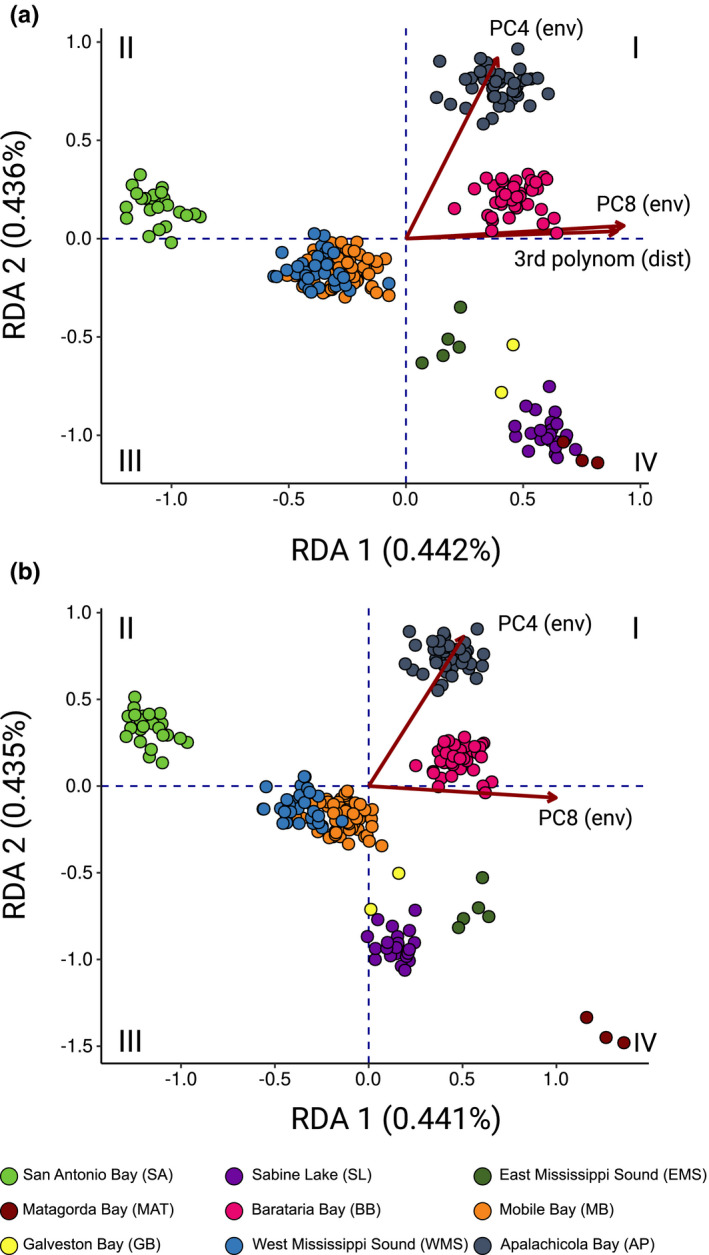
Biplot of redundancy analysis using (a) a full model using explanatory matrices selected for the best spatial model (3rd degree polynomial of coastal distance) and environmental model (PC8, PC4) and (b) the environmental model alone. Individuals sampled in the Gulf (colored circles) are plotted according to their component loadings calculated as weighted average scores. The full model explains 1.3% of variance, RDA1 and 2 explain 33.95% (0.442%) and 33.40% (0.436%) of constrained (total) variance, respectively. The environmental model explains 0.8% of variance, RDA1 and 2 explain 50.31% (0.441%) and 49.69% (0.435%) of constrained (total) variance. Quadrants I–IV are labeled for easier reference in the text

Clustering of individuals was compared for the full model and environmental model only (Figure 5). A biplot of the full RDA model (Figure 5a) revealed a complex pattern with individuals from the same estuary forming clusters, but with placement of individual estuaries along individual RDA axes not always corresponding to geographic proximity. San Antonio Bay and Mobile Bay and West Mississippi Sound formed two clusters separated from other samples along RDA 1 (Quadrant II, III) driven by the spatial matrix and PC 8. Individuals from the remaining estuaries were resolved by RDA 2, with Apalachicola Bay and Barataria Bay forming two distinct clusters in Quadrant I and East Mississippi Sound, Galveston Bay and Sabine Lake/Matagorda Bay individuals forming clusters in Quadrant IV. The environmental model clustered individuals in a very similar pattern (Figure 5b), the difference being how the clusters lie relative to each other in Quadrant IV. Sabine Lake and Matagorda Bay individuals clustered separately, and Sabine Lake and Galveston Bay individuals were closer to the Mobile Bay/West Mississippi Sound cluster, while Matagorda remained in the lower right‐hand corner of Quadrant IV. Alleles at 384 loci (9.3%) were flagged as strongly associated with both RDA axis of the environmental model (Mahalanobis Distance >25). Of these, 123 had been previously mapped on the linkage map and appeared to be randomly distributed across all chromosomes (Figure S1).

## 4 DISCUSSION

Here, reduced representation sequencing was used to identify patterns of contemporary population structure of southern flounder sampled in the U.S. Atlantic and Gulf of Mexico. Significant divergence was observed between the Atlantic and Gulf. While amova based on neutral markers indicated significant heterogeneity among estuaries within the Gulf, most pairwise comparisons of sampled estuaries within ocean basins were not significant. Overall, Gulf estuaries exhibited significantly higher levels of allelic diversity as compared with the Atlantic estuaries, though no explicit spatial patterns of within‐sample diversity were detected within the Gulf and estuaries in both basins exhibited mutation‐drift disequilibrium at genome‐wide scales. Landscape genomic approaches revealed a pattern of nonlinear isolation by distance across the northern Gulf and indicated that environmental variables summarizing differences among individual estuaries explain a larger portion of genomic variation than geographic distance.

Significant divergence between Atlantic and Gulf populations of southern flounder aligns with a well‐documented biogeographic break associated with southern peninsular Florida (Neigel, 2009). Genetic discontinuities associated with southern Florida have been observed in a number of nearshore and estuarine species (Gold & Richardson, 1998) and are likely related to a lack of appropriate estuarine habitat. Consistent with this, southern flounder show a gap in their distribution in southern Florida, approximately between Tampa and Cape Canaveral (Ginsburg, 1952). Given adult movement ecology in the Gulf and Atlantic, larval dispersal, and the results here, it seems highly unlikely that the Atlantic and Gulf experience biologically important levels of contemporary gene flow.

*F*_ST_‐outlier loci were detected when analyzing Gulf and Atlantic samples together. By contrast, no outlier loci were detected when Atlantic and Gulf data sets were analyzed separately. *F*_ST_‐outliers are generally considered to be under positive selection, though historical demographic processes may create similar patterns (Lotterhos & Whitlock, 2014). Here, analysis of Tajima's *D* indicated a genome‐wide departure from mutation‐drift equilibrium consistent with population expansion, rather than departures at specific loci only, which would be consistent with directional selection. This mirrors other studies that have detected postglacial expansion in marine species from the Gulf and U.S. South Atlantic (Marko & Hart, 2012; Portnoy et al., 2014). Furthermore, loci flagged as *F*_ST_‐outliers or with significantly negative Tajima's *D* (both taken to indicate directional selection) were spread across the genome rather than clustered together, the latter expected if strong, locus‐specific directional selection was present (Nielsen, 2001). Simulation studies have shown that differentiation‐based outlier tests are not robust when applied to nonequilibrium demographic scenarios, specifically isolation‐by‐distance and range expansions, and may suffer increased rates of false positives (Forester et al., 2018; Hoban et al., 2016; Lotterhos & Whitlock, 2014). While directional selection operating at an ocean basin scale cannot be ruled out, the agreement in pattern between putatively neutral loci and outlier loci along with evidence of recent expansion, suggest that *F*_ST_ outlier‐loci identified in this study are drift outliers.

Traditional *F*_ST_‐based approaches assume that microevolutionary forces are in equilibrium and simple demographic models, such as island or stepping stone models, are sufficient to explain observed patterns of genetic heterogeneity (Holsinger & Weir, 2009). When these assumptions are violated, which is the case for many marine species (Waples, 1998), spatially explicit analyses are preferable (Manel et al., 2003). For southern flounder within the Gulf, pairwise estimates of *F*_ST_ between estuaries were generally not significant, despite global tests indicating significant genetic heterogeneity across the Gulf. By contrast, RDA indicated spatial autocorrelation and a pattern of nonlinear isolation by distance among estuaries in the Gulf. Further, environmental similarities (or differences) among habitats explained a significant component of total genomic variation that was larger than geographic distance, even though *F*_ST_ outlier tests found no significant departure from neutral expectations in the Gulf. Environmental conditions may change allele frequencies in concert with or in opposition to drift processes (Kawecki & Ebert, 2004), and the latter seems to have occurred in southern flounder. For example, individuals from estuaries in the western Gulf do not cluster together when constraining the variation to the component explained by the full RDA model (Figure 5a). Furthermore, the position of the Sabine Lake cluster relative to other estuaries changes when constraining the variance to the component explained by the environmental model (Figure 5b). The shift in how individuals from estuaries in Quadrant IV cluster relative to each other, depending on whether or not spatial signal is explicitly accounted for, has real world applications. Currently, southern flounder are part of a stock‐enhancement program in the western Gulf where captive wild‐caught adults are spawned in a hatchery and YOY head‐started in outdoor ponds. The extent to which YOY should be returned to the estuaries from which their parents were caught or whether they can be efficiently stocked in neighboring estuaries has remained an open question (Kaiser et al., 2012; Miller et al., 2010). The results present here indicate that it is important to consider environmental differences among estuaries as well as geographic proximity.

While the full RDA model only explained a small percentage of total variation (approx. 1%), with the majority attributable to environment, the results are not that dissimilar from other studies. Bay et al. (2017) presented a review of genomic studies and found that between 0.002% and 4.6% of loci, across studies, appeared to be influenced by environment. Furthermore, gene flow across estuaries combined with selection operating on multiple genes but of small effect can result in a swamping of the signal (Yeaman, 2015). A similar result was seen in a study of the congeneric summer flounder*, Paralichthys dentatus,* in the Atlantic, where environmental factors appear to drive small but important differences in genetic diversity despite near‐panmixia and no evidence of significant population structure across most of the genome (Hoey & Pinsky, 2018).

For southern flounder, spawning takes place offshore from nursery areas and prevailing currents within and between years determine the strength and sources of larval influx, though habitat quality and availability for settling may ultimately dictate recruitment (Burke et al., 1991; Miller et al., 1991). Consistent with this, environmental variables of importance identified using the RDA framework included tidal influence and freshwater input, factors impacting differences in salinity among and within estuaries, as well as available wetland habitat. These results parallel studies that have identified environmental variables, including estuary depth and slope, tidal height, habitat type, proximity to inlet, temperature, salinity, turbidity, and levels of dissolved oxygen, as determinates of the density of juvenile southern flounder within estuaries (Glass et al., 2008; Nañez‐James et al., 2009). Further, these studies indicate that preferences for combinations of conditions seem to vary across estuaries. For example, juvenile southern flounder in estuaries off North Carolina were usually sampled in shallow water with low salinity, high dissolved oxygen, and muddy bottom substrates, and found far from the estuary inlet (Burke et al., 1991; Powell & Schwartz, 1977); while in Aransas Bay (Texas), juvenile abundance was highest in vegetated, sandy areas with higher salinities and located near estuary inlets, with low abundances in muddy bottoms habitat (Nañez‐James et al., 2009). Additionally, estuaries are a mosaic of microhabitats and juvenile southern flounder exhibit ontogenetic shifts in microhabitat use (Amorim et al., 2018; Furey & Rooker, 2013; Miller et al., 1991). In the Gulf, juvenile southern flounder initially settle into structurally complex habitats like seagrass beds and marsh edges, transitioning to habitats characterized by sandy or muddy substrates toward the end of their juvenile stages (Furey & Rooker, 2013; Glass et al., 2008; Nañez‐James et al., 2009). Access to appropriate habitat during development may strongly affect survival of juveniles at a time when mortality rates are high and the availability and types of habitat differ among estuaries (Amorim et al., 2018; Burke et al., 1991). This sets up a complex scenario in which gene flow mediated by larval, and perhaps adult, dispersal is related to distance, while environmental and physiochemical conditions that vary among and within estuaries dictate survival of recruits (Burke et al., 1998). This results in components of genetic diversity shaped initially by dispersal (migration and drift), and subsequently refined by characteristics of the local habitat experienced by juveniles (selection) in such a way that there may be conflicting signal within the genome. Estuaries by their nature are dynamic habitats that vary environmentally across years and such a dynamic could lead to an association between genetic variation and interannual environmental and climate variability but would require temporally explicit samples across cohorts to detect, which was not possible in this study.

Additionally, environmental heterogeneity and ontogenetic shifts in habitat use may facilitate the maintenance of diversity (balancing selection), rather than favor specific phenotypes (directional/purifying selection), when survivorship is determined by genotype‐environment matches at the microhabitat scale (Bernatchez et al., 2019; Marshall et al., 2010). The complex interactions of competing evolutionary forces, along with diversifying or weak polygenic selection can result in genetic diversity characterized by the presence of rare alleles (Kawecki & Ebert, 2004; Star & Spencer, 2013), as seen in southern flounder samples in the Gulf. Consistent with this idea, loci with large Mahalanobis distance (>25) were spread throughout the genome, rather than grouped, and there were no significant *F*_ST_‐outlier loci in the Gulf indicative of strong directional selection. Furthermore, patterns of reduced genetic diversity in the Atlantic relative to the Gulf are largely attributable to a reduction in the prevalence of rare alleles and could be due to less environmental heterogeneity, but also stronger genetic drift and/or a smaller source population postglacial expansion (Allendorf, 1986). While the exact mechanisms cannot be inferred due to limited sampling in the Atlantic, estimates of Θ_W_ and Θ_π_ were both smaller in Atlantic estuaries than the Gulf estuaries, suggesting that reduced long‐term effective population size in the Atlantic may at least partially explain the pattern. The presence of multiple forces shaping genomic diversity, to an extent in opposing ways, likely contributes to distinct signals parsed in this study each only explaining small though significant proportions of total variance (Yeaman, 2015).

Understanding the interplay of microevolutionary processes has important implications for marine species, especially for those like southern flounder that are the focus of commercial/recreation fisheries and stock augmentation programs. A pattern of high gene flow and isolation by distance alone would suggest that the geographic origin of fish might matter only on large spatial scales for stocking purposes. By contrast, the finding that environmental differences among estuaries explains total genomic variation better than relative geographic position indicates that best management strategies for southern flounder should include a focus on the preservation of a diversity of habitats that can be used to complete early life stages. Ecosystem‐based management approaches already emphasize the preservation of critical habitat to sustain populations but often argue from a standpoint of the importance of certain specific habitats (Rosenberg et al., 2000). In this study, environmental–genome associations were related to relative differences (or similarities) in the environment among estuaries, and environmental characteristics of particular estuaries explained a significant but small proportion (approx. 1%) of genomic variation among estuaries. The observations that the environmental conditions that contribute to survival and successful reproduction (fitness) vary in space suggests that not only the availability of specific habitats but also the diversity of available habitat types is important. Further, in southern flounder selection appears to be complex, involving many loci spread throughout the genome, and the results add to a growing body of research that demonstrates the importance of considering models of gene–environment interactions without equilibrium assumptions, rather than only screening for *F*_ST_‐based outlier loci indicative of strong directional selection (Forester et al., 2018; Hoban et al., 2016).

## CONFLICT OF INTEREST

The authors declare no competing interests.

## AUTHOR CONTRIBUTIONS

**Shannon J. O'Leary:** Conceptualization (supporting); Data curation (lead); Formal analysis (lead); Methodology (lead); Project administration (lead); Visualization (lead); Writing‐original draft (lead); Writing‐review & editing (equal). **David S. Portnoy:** Conceptualization (lead); Formal analysis (supporting); Investigation (supporting); Methodology (supporting); Project administration (supporting); Resources (lead); Writing‐review & editing (equal). **Christopher M. Hollenbeck:** Formal analysis (supporting); Methodology (supporting); Writing‐review & editing (equal). **Robert R. Vega:** Formal analysis (supporting); Methodology (supporting); Writing‐review & editing (equal).

## Supporting information

Figure S1

Figure S2

Figure S3

Tables S1‐S4

## Data Availability

Raw reads are submitted at the short reads archive (BioProject PRJNA754841). Processed data sets underlying the analysis, custom scripts, and R markdown files with reproducible code are available at https://github.com/sjoleary/SFL_PopGen.
