# Supplementary material for: Disentangling complex genomic signals to understand population structure of an exploited, estuarine‐dependent flatfish
Source: Ecol Evol. 2021 Aug 30;11(19):13415–29. doi: 10.1002/ece3.8064 (PMC8495835; doi:10.1002/ece3.8064)
Supplement: Supplementary file 4 — Tables S1‐S4 [file ECE3-11-13415-s003.docx]

# Appendix

Appendix Table S1: Pairwise comparisons of (A) gene diversity, (B) rarefied allele counts, and (C) evenness among estuaries. Friedman’s test indicated significant heterogeneity for all three measures of genetic diversity. Significance of pairwise differences was assessed using Wilcoxon signed rank test. The test statistic Q and level of significance before (P) and after (P adjusted) correction are indicated along with whether the comparison of estuaries is between ocean basins or within.

**A**

| **Estuaries** |  | ***Q*** | ***P*** | ***P* adjusted** | **Comparison** |
| --- | --- | --- | --- | --- | --- |
| Winyah Bay | St Johns River | 0.00 | 0.99938 | 0.99938 | Atl/Atl |
| Apalachicola Bay | Mobile Bay | 0.07 | 0.94052 | 0.96739 | Gulf/Gulf |
| San Antonio Bay | Apalachicola Bay | 0.26 | 0.79390 | 0.84060 | Gulf/Gulf |
| Apalachicola Bay | Barataria Bay | 0.57 | 0.56670 | 0.61822 | Gulf/Gulf |
| Mobile Bay | Barataria Bay | 0.66 | 0.50644 | 0.56974 | Gulf/Gulf |
| Barataria Bay | West Mississippi Sound | 0.88 | 0.37688 | 0.43767 | Gulf/Gulf |
| San Antonio Bay | Barataria Bay | 0.99 | 0.31997 | 0.38941 | Gulf/Gulf |
| San Antonio Bay | Mobile Bay | 0.99 | 0.32450 | 0.38941 | Gulf/Gulf |
| Charleston Harbor | Winyah Bay | 1.11 | 0.26723 | 0.34358 | Atl/Atl |
| Apalachicola Bay | West Mississippi Sound | 1.46 | 0.14337 | 0.19116 | Gulf/Gulf |
| Mobile Bay | West Mississippi Sound | 1.55 | 0.12196 | 0.16886 | Gulf/Gulf |
| Charleston Harbor | St Johns River | 1.73 | 0.08397 | 0.12092 | Atl/Atl |
| San Antonio Bay | West Mississippi Sound | 2.02 | 0.04308 | 0.06462 | Gulf/Gulf |
| West Mississippi Sound | Charleston Harbor | 2.73 | 0.00640 | 0.01002 | Gulf/Atl |
| West Mississippi Sound | St Johns River | 2.84 | 0.00455 | 0.00745 | Gulf/Atl |
| Sabine Lake | San Antonio Bay | 2.92 | 0.00352 | 0.00603 | Gulf/Gulf |
| Barataria Bay | Charleston Harbor | 2.98 | 0.00291 | 0.00524 | Gulf/Atl |
| Mobile Bay | Charleston Harbor | 3.48 | 0.00050 | 0.00095 | Gulf/Atl |
| Apalachicola Bay | Charleston Harbor | 3.48 | 0.00050 | 0.00095 | Gulf/Atl |
| San Antonio Bay | Charleston Harbor | 3.61 | 0.00030 | 0.00064 | Gulf/Atl |
| Barataria Bay | St Johns River | 3.80 | 0.00015 | 0.00033 | Gulf/Atl |
| Sabine Lake | Apalachicola Bay | 3.81 | 0.00014 | 0.00033 | Gulf/Gulf |
| West Mississippi Sound | Winyah Bay | 3.95 | 0.00008 | 0.00020 | Gulf/Atl |
| Sabine Lake | Mobile Bay | 3.98 | 0.00007 | 0.00019 | Gulf/Gulf |
| Apalachicola Bay | St Johns River | 4.12 | 0.00004 | 0.00012 | Gulf/Atl |
| Mobile Bay | St Johns River | 4.17 | 0.00003 | 0.00010 | Gulf/Atl |
| Barataria Bay | Winyah Bay | 4.28 | 0.00002 | 0.00007 | Gulf/Atl |
| Sabine Lake | Barataria Bay | 4.34 | 0.00001 | 0.00006 | Gulf/Gulf |
| San Antonio Bay | St Johns River | 4.35 | 0.00001 | 0.00006 | Gulf/Atl |
| San Antonio Bay | Winyah Bay | 4.94 | 0.00000 | 0.00000 | Gulf/Atl |
| Apalachicola Bay | Winyah Bay | 5.11 | 0.00000 | 0.00000 | Gulf/Atl |
| Mobile Bay | Winyah Bay | 5.19 | 0.00000 | 0.00000 | Gulf/Atl |
| Sabine Lake | West Mississippi Sound | 5.24 | 0.00000 | 0.00000 | Gulf/Gulf |
| Sabine Lake | Charleston Harbor | 5.68 | 0.00000 | 0.00000 | Gulf/Atl |
| Sabine Lake | St Johns River | 5.93 | 0.00000 | 0.00000 | Gulf/Atl |
| Sabine Lake | Winyah Bay | 6.35 | 0.00000 | 0.00000 | Gulf/Atl |

B

| **Estuaries** |  | ***Q*** | ***P*** | ***P* adjusted** | **Comparison** |
| --- | --- | --- | --- | --- | --- |
| Apalachicola Bay | Mobile Bay | 0.05 | 0.95652 | 0.95652 | Gulf/Gulf |
| St Johns River | Winyah Bay | 0.27 | 0.78655 | 0.80903 | Atl/Atl |
| San Antonio Bay | West Mississippi Sound | 0.67 | 0.49982 | 0.52922 | Gulf/Gulf |
| Barataria Bay | San Antonio Bay | 0.81 | 0.41988 | 0.45805 | Gulf/Gulf |
| Mobile Bay | Barataria Bay | 0.88 | 0.37780 | 0.42502 | Gulf/Gulf |
| Apalachicola Bay | Barataria Bay | 1.26 | 0.20901 | 0.24272 | Gulf/Gulf |
| Mobile Bay | San Antonio Bay | 1.92 | 0.05451 | 0.06541 | Gulf/Gulf |
| Apalachicola Bay | San Antonio Bay | 1.97 | 0.04914 | 0.06100 | Gulf/Gulf |
| Barataria Bay | West Mississippi Sound | 2.07 | 0.03887 | 0.04998 | Gulf/Gulf |
| Apalachicola Bay | West Mississippi Sound | 2.85 | 0.00436 | 0.00581 | Gulf/Gulf |
| Charleston Harbor | Winyah Bay | 3.03 | 0.00245 | 0.00339 | Atl/Atl |
| Charleston Harbor | St Johns River | 3.09 | 0.00197 | 0.00284 | Atl/Atl |
| Mobile Bay | West Mississippi Sound | 3.21 | 0.00133 | 0.00199 | Gulf/Gulf |
| Sabine Lake | Apalachicola Bay | 3.85 | 0.00012 | 0.00018 | Gulf/Gulf |
| Sabine Lake | Mobile Bay | 4.43 | 0.00001 | 0.00002 | Gulf/Gulf |
| Sabine Lake | Barataria Bay | 4.92 | 0.00000 | 0.00000 | Gulf/Gulf |
| Sabine Lake | San Antonio Bay | 5.10 | 0.00000 | 0.00000 | Gulf/Gulf |
| Sabine Lake | West Mississippi Sound | 6.27 | 0.00000 | 0.00000 | Gulf/Gulf |
| West Mississippi Sound | Charleston Harbor | 17.79 | 0.00000 | 0.00000 | Gulf/Atl |
| San Antonio Bay | Charleston Harbor | 17.85 | 0.00000 | 0.00000 | Gulf/Atl |
| Barataria Bay | Charleston Harbor | 18.82 | 0.00000 | 0.00000 | Gulf/Atl |
| San Antonio Bay | St Johns River | 18.92 | 0.00000 | 0.00000 | Gulf/Atl |
| West Mississippi Sound | St Johns River | 19.04 | 0.00000 | 0.00000 | Gulf/Atl |
| San Antonio Bay | Winyah Bay | 19.90 | 0.00000 | 0.00000 | Gulf/Atl |
| Apalachicola Bay | Charleston Harbor | 20.03 | 0.00000 | 0.00000 | Gulf/Atl |
| West Mississippi Sound | Winyah Bay | 20.08 | 0.00000 | 0.00000 | Gulf/Atl |
| Mobile Bay | Charleston Harbor | 20.26 | 0.00000 | 0.00000 | Gulf/Atl |
| Barataria Bay | St Johns River | 20.45 | 0.00000 | 0.00000 | Gulf/Atl |
| Sabine Lake | Charleston Harbor | 20.81 | 0.00000 | 0.00000 | Gulf/Atl |
| Apalachicola Bay | St Johns River | 21.34 | 0.00000 | 0.00000 | Gulf/Atl |
| Barataria Bay | Winyah Bay | 21.40 | 0.00000 | 0.00000 | Gulf/Atl |
| Mobile Bay | St Johns River | 21.79 | 0.00000 | 0.00000 | Gulf/Atl |
| Sabine Lake | St Johns River | 22.17 | 0.00000 | 0.00000 | Gulf/Atl |
| Apalachicola Bay | Winyah Bay | 22.33 | 0.00000 | 0.00000 | Gulf/Atl |
| Mobile Bay | Winyah Bay | 22.75 | 0.00000 | 0.00000 | Gulf/Atl |
| Sabine Lake | Winyah Bay | 22.82 | 0.00000 | 0.00000 | Gulf/Atl |

C

| **Estuaries** |  | ***Q*** | ***P*** | ***P* adjusted** | **Comparison** |
| --- | --- | --- | --- | --- | --- |
| Winyah Bay | Charleston Harbor | 1.35 | 0.17690 | 0.17690 | Atl/Atl |
| Charleston Harbor | St Johns River | 2.40 | 0.01653 | 0.01700 | Atl/Atl |
| Barataria Bay | Apalachicola Bay | 3.38 | 0.00073 | 0.00078 | Gulf/Gulf |
| Winyah Bay | St Johns River | 3.52 | 0.00042 | 0.00046 | Atl/Atl |
| San Antonio Bay | Sabine Lake | 4.43 | 0.00001 | 0.00001 | Gulf/Gulf |
| West Mississippi Sound | Barataria Bay | 5.13 | 0.00000 | 0.00000 | Gulf/Gulf |
| Sabine Lake | West Mississippi Sound | 8.41 | 0.00000 | 0.00000 | Gulf/Gulf |
| West Mississippi Sound | Apalachicola Bay | 8.79 | 0.00000 | 0.00000 | Gulf/Gulf |
| Apalachicola Bay | Mobile Bay | 9.53 | 0.00000 | 0.00000 | Gulf/Gulf |
| Barataria Bay | Mobile Bay | 12.42 | 0.00000 | 0.00000 | Gulf/Gulf |
| San Antonio Bay | West Mississippi Sound | 12.52 | 0.00000 | 0.00000 | Gulf/Gulf |
| Sabine Lake | Barataria Bay | 12.75 | 0.00000 | 0.00000 | Gulf/Gulf |
| Sabine Lake | Apalachicola Bay | 15.66 | 0.00000 | 0.00000 | Gulf/Gulf |
| St Johns River | San Antonio Bay | 17.23 | 0.00000 | 0.00000 | Atl/Gulf |
| San Antonio Bay | Barataria Bay | 17.40 | 0.00000 | 0.00000 | Gulf/Gulf |
| West Mississippi Sound | Mobile Bay | 17.57 | 0.00000 | 0.00000 | Gulf/Gulf |
| Charleston Harbor | San Antonio Bay | 19.40 | 0.00000 | 0.00000 | Atl/Gulf |
| San Antonio Bay | Apalachicola Bay | 19.89 | 0.00000 | 0.00000 | Gulf/Gulf |
| Winyah Bay | San Antonio Bay | 19.99 | 0.00000 | 0.00000 | Atl/Gulf |
| St Johns River | Sabine Lake | 20.15 | 0.00000 | 0.00000 | Atl/Gulf |
| Charleston Harbor | Sabine Lake | 21.79 | 0.00000 | 0.00000 | Atl/Gulf |
| Winyah Bay | Sabine Lake | 22.65 | 0.00000 | 0.00000 | Atl/Gulf |
| Sabine Lake | Mobile Bay | 23.81 | 0.00000 | 0.00000 | Gulf/Gulf |
| St Johns River | West Mississippi Sound | 24.84 | 0.00000 | 0.00000 | Atl/Gulf |
| Charleston Harbor | West Mississippi Sound | 26.42 | 0.00000 | 0.00000 | Atl/Gulf |
| Winyah Bay | West Mississippi Sound | 26.85 | 0.00000 | 0.00000 | Atl/Gulf |
| San Antonio Bay | Mobile Bay | 26.88 | 0.00000 | 0.00000 | Gulf/Gulf |
| St Johns River | Barataria Bay | 27.20 | 0.00000 | 0.00000 | Atl/Gulf |
| St Johns River | Apalachicola Bay | 28.52 | 0.00000 | 0.00000 | Atl/Gulf |
| Charleston Harbor | Barataria Bay | 28.59 | 0.00000 | 0.00000 | Atl/Gulf |
| Winyah Bay | Barataria Bay | 29.19 | 0.00000 | 0.00000 | Atl/Gulf |
| Charleston Harbor | Apalachicola Bay | 30.11 | 0.00000 | 0.00000 | Atl/Gulf |
| Winyah Bay | Apalachicola Bay | 30.54 | 0.00000 | 0.00000 | Atl/Gulf |
| St Johns River | Mobile Bay | 31.82 | 0.00000 | 0.00000 | Atl/Gulf |
| Charleston Harbor | Mobile Bay | 33.25 | 0.00000 | 0.00000 | Atl/Gulf |
| Winyah Bay | Mobile Bay | 33.80 | 0.00000 | 0.00000 | Atl/Gulf |

Appendix Table S2: Comparison of observed mean and median values for Tajima’s D compared to simulated data sets in mutation-drift equilibrium. For all estuaries, the observed values were significantly lower compared to the simulated data set.

| **Estuary** |  | **Observed** | **Simulated** |
| --- | --- | --- | --- |
| San Antonio Bay | mean | -0.6312303 | -0.0565064 |
|  | median | -0.8235974 | -0.1782294 |
| Sabine Lake | mean | -0.6889841 | -0.0786213 |
|  | median | -0.8621855 | -0.1846628 |
| Barataria Bay | mean | -0.6707489 | -0.0633617 |
|  | median | -0.8441499 | -0.1790930 |
| West Mississippi Sound | mean | -0.6647244 | -0.0667930 |
|  | median | -0.8347845 | -0.1894230 |
| Mobile Bay | mean | -0.6717212 | -0.0695248 |
|  | median | -0.8432089 | -0.1896403 |
| Apalachicola Bay | mean | -0.6661067 | -0.0890480 |
|  | median | -0.8291125 | -0.1978763 |
| St. Johns River | mean | -0.3462437 | -0.0439707 |
|  | median | -0.3462437 | -0.6667912 |
| Charleston Harbor | mean | -0.3696008 | -0.0603542 |
|  | median | -0.5129384 | -0.1666273 |
| Winyah Bay | mean | -0.3282842 | -0.0601895 |
|  | median | -0.4826751 | -0.1648431 |

Appendix Table S3: Distribution of mean +/- standard deviation (std) of Watterson’s estimator Θ_W_ across sampled estuaries with > 18 individuals.

| **Estuary** | **mean** | **std** |
| --- | --- | --- |
| San Antonio Bay | 0.4543 | 0.3537 |
| Sabine Lake | 0.4814 | 0.3642 |
| Barataria Bay | 0.5364 | 0.3970 |
| West Mississippi Sound | 0.5122 | 0.3805 |
| Mobile Bay | 0.6024 | 0.4263 |
| Apalachicola Bay | 0.5515 | 0.4013 |
| St Johns River | 0.3704 | 0.3042 |
| Charleston Harbor | 0.3596 | 0.2960 |
| Winyah Bay | 0.3531 | 0.2952 |

Appendix Table S4: Distribution of mean +/- standard deviation (std) of Watterson’s estimator Θ_T_ across sampled estuaries with > 18 individuals.

| **pop** | **mean** | **std** |
| --- | --- | --- |
| San Antonio Bay | 0.0021 | 0.0019 |
| Sabine Lake | 0.0021 | 0.0019 |
| Barataria Bay | 0.0021 | 0.0019 |
| West Mississippi Sound | 0.0021 | 0.0019 |
| Mobile Bay | 0.0021 | 0.0019 |
| Apalachicola Bay | 0.0021 | 0.0019 |
| St Johns River | 0.0020 | 0.0019 |
| Charleston Harbor | 0.0020 | 0.0019 |
| Winyah Bay | 0.0020 | 0.0019 |

Appendix Figure S1: Distribution of (A) F_ST_, by locus position for loci previously mapped on a Southern Flounder linkage map. Loci flagged as neutral or not significantly associated are in grey, loci flagged as outlier or significantly associated with environmental variables are indicated in red.

Appendix Figure S2: Distribution of Tajima’s D for individuals grouped by estuary by locus position for loci previously mapped on a Southern Flounder linkage map. Loci flagged as neutral are in grey, loci flagged as outlier are indicated in red. AP = Apalachicola Bay, BB = Barataria Bay, CH = Charleston Harbor, MB = Mobile Bay, SA = San Antonio Bay, SJR = St John’s River, SL = Sabine Lake, WB = Winyah Bay, WMS = West Mississippi Sound.

Appendix Figure S3: Distribution of Mahalanobis distance by locus position for loci previously mapped on a Southern Flounder linkage map. Loci flagged as not significantly associated are in grey, loci flagged as significantly associated with environmental variables are indicated in red.
